# Lactoferrin Functionalized Biomaterials: Tools for Prevention of Implant-Associated Infections

**DOI:** 10.3390/antibiotics9080522

**Published:** 2020-08-15

**Authors:** Emoke Pall, Alexandra Roman

**Affiliations:** 1 Life Science Institute, University of Agricultural Sciences and Veterinary Medicine, Cluj-Napoca 400372, Romania; 2 Department of Periodontology, Faculty of Dental Medicine, Iuliu Haţieganu University of Medicine and Pharmacy, Cluj-Napoca 400012, Romania; alexandra.roman@umfcluj.ro

**Keywords:** implant-associated infection, surface treatment, antimicrobial peptides, lactoferrin, antimicrobials, prevention

## Abstract

Tissue engineering is one of the most important biotechnologies in the biomedical field. It requires the application of the principles of scientific engineering in order to design and build natural or synthetic biomaterials feasible for the maintenance of tissues and organs. Depending on the specific applications, the selection of the proper material remains a significant clinical concern. Implant-associated infection is one of the most severe complications in orthopedic implant surgeries. The treatment of these infections is difficult because the surface of the implant serves not only as a substrate for the formation of the biofilm, but also for the selection of multidrug-resistant bacterial strains. Therefore, a promising new approach for prevention of implant-related infection involves development of new implantable, non-antibiotic-based biomaterials. This review provides a brief overview of antimicrobial peptide-based biomaterials—especially those coated with lactoferrin.

## 1. Introduction

Degenerative and inflammatory problems of bone and joint affect millions of people worldwide and represents one-half of all chronic diseases that affect people over the age of 50 in developed countries [1,2]. These conditions often require surgery and transplantation of permanent, temporary or biodegradable devices [1] with structural and surface compatibility with the host tissue [2]. Biomaterials can be used in medical applications to treat, regenerate or replace any tissue, organ or function of the body [3,4]. Three generation of material have been used for biomedical purpose: bioinert materials; bioactive and biodegradable; and materials with ability to stimulate specific cellular responses at the molecular level [1,5]. Biomaterials should possess good mechanical, physical, chemical and biologic properties, biocompatibility [6,7,8] and antimicrobial properties to overcome implant-associated infections [9]. Depending on the specific application, selection of the proper material for orthopedic implants is essential. Orthopedic implants are classified into two categories: permanent joint replacements and temporary fracture-fixation equipment [8]. Metals and their alloys (Ti–6Al–4V, Co–Cr–Mo and stainless steel), ceramics (alumina, zirconia and hydroxyapatite) and biocomposites are commonly used in orthopedic implants [2,10,11].

Polymers are considered alternative materials to conventional metallic equivalent in orthopedics [12]. The design of these biomaterials is done in a manner that they can stimulate certain biologic responses or can promote bone tissue adhesion [5,11]. A large part of these biomaterials are biodegradable, and recently their surfaces have been functionalized with bioactive molecules for liberate at pathologic sites [11,13,14]. The important properties of biodegradable biomaterial include characteristics related to their mechanical property, permeability, cytotoxicity, resistance and degradation [4,15]. The mechanical features of the biomaterial should be adequate to promote tissue regeneration. The degradation time should coincide with the regeneration or with the healing process [16]. This type of biomaterials can be classified as synthetic, natural or biologically derived and inorganic polymers [4,17] and their mechanical properties are similar to tissues. Synthetic biopolymers have several advantages compared with the natural biopolymers such as mechanical solubility and morphologic properties [18].

Natural polymeric biomaterials usually include proteins and polysaccharides [19,20], chitosan, hyaluronic acid, chondroitin sulfate, collagen and fibrin [11]. Recently, polyhydroxyalkanoates (PHA), a native polyesters, natural polysaccharide-based hydrogels and ivy nanoparticles have been recognized as natural biodegradable biomaterials [18]. Natural polymers possess several intrinsic advantages such as natural remodeling and a specific capacity to present receptor-binding ligands to cells and also susceptibility to proteolytic degradation—but also downsides, such as strong immunogenic response after transplantation [4]. Synthetic biomaterials are generally biologically inert; these materials can be modified to hold a large of mechanical and chemical properties for specific applications [16]. Synthetic biofunctional materials include polyglycolide (PGA), polylactide (PLA), polydioxanone (PDO), poly(2hydroxyethylmethacrylate) (PHEMA), poly(e-caprolactone) (PCL), ultrahigh molecular weight polyethylene (UHMWPE), poly(methylmethacrylate) (PMMA), polyurethanes (PU), polyether ether ketone (PEEK), polyorthoesters and hydrogels [11,12]. Degradable polymeric biomaterials are good candidates for developing new therapeutic devices such as temporary prostheses, scaffolds for tissue engineering and they are also controlled/sustained release drug delivery vehicles [4,16]. The bioactivity of polymers depends on the functional groups and the material surface. The bioactivity can be improved by surface modification with biomolecules [1,21,22]. The third generation of biomaterials is new materials able to stimulate specific cellular responses at the molecular level [5] characterized by bioactivity and biodegradability. These biomaterials are three-dimensional porous structures able to stimulate cells invasion, attachment and proliferation [1,23].

## 2. Implant-Associated Infections

Implant-associated infections are a major problem in modern medicine, despite continuous improvements in device design, surgical procedures and wound care [24]. Bacterial infection following trauma and orthopedic implant surgery remain the most severe complications [2,25,26], associated with prolonged morbidity, disability and increased mortality [27]. The rate of infection associated with such surgeries is approximately 0.8%−1.2% for total hip arthroplasty, 2% for primary joint replacements and about 14% of the total hip and knee revisions due to infection and in terms of trauma surgery 3.6%−8.1% after closed fractures, 17.5%−21.2% after open fractures [25,26,28,29]. In implant-associated infection several factors are involved such as surgical procedure, microorganisms, host, type of the implant and antimicrobial prophylaxis [30,31]. The predominant microbial agent isolated from implant-associated infection is *Staphylococcus aureus* (30%), followed by coagulase-negative staphylococci (22%), but Gram-negative bacteria, enterococci, streptococci and other species may be isolated [32,33]. In conformity with current knowledge, biofilm formation presumably is the most critical pathogenic event in implant-associated infection. The implants protect the microorganisms from the host immune system and systemic antibiotics [30]. Bacterial adhesion to the implant surface—and colonization of the tissue surrounding the implant secretion of exopolysaccharides, aggregation in a slime layer and further differentiation and biofilm formation—are the most significant steps in implant infection [2,32,33,34,35]. Bacterial adhesion to the surfaces is generally nonspecific and it is produced by unspecific forces, namely, van der Wall, acid base or electrostatic interaction [36]. On medical devices, both Gram-positive and Gram-negative bacteria have the ability to form biofilms, but most often *Enterococcus fecalis*, *Staphylococcus aureus*, *Staphylococcus epidermidis*, *Streptococcus viridans*, *E. coli*, *Klebsiella pneumoniae*, *Proteus mirabilis* and *Pseudomonas aeruginosa* [37,38] have been identified. Implant infection depend on the type, place and time of the intervention. Early infections occur during surgery; usually virulent *Staphylococcus* spp. is involved. Late infections are the result of implant colonization by low-virulence microorganisms, which reach the site via hematogenous dissemination from skin, dental, oropharyngeal and urinary tract infections [2].

The incidence of multidrug-resistant Gram-negative (*Escherichia coli, Klebsiella pneumoniae, Proteus mirabilis* and *Pseudomonas aeruginosa,* multidrug-resistant *Acinetobacter*) microorganism involved in implant related infections is of around 8% [35,39,40]. Gram-positive *Enterococcus fecalis*, *Streptococcus pyogenes*, *Staphylococcus aureus,* methicillin and vancomycin-resistant *Staphylococcus aureus*, *Staphylococcus epidermidis*, *Corynebacterium* spp., *Propionibacterium acnes*, *Peptococcus saccharides*, *Peptococcus magnus*, *Peptostreptococcus magnus*, *Enterobacter* species and *Streptococcus viridan*s are some of the bacterial strains commonly associated with implant-associated infection [26,35]. Effective antimicrobial treatment of these pathologies is difficult because the surface of the implant serves as a substrate for the formation of biofilms [41,42,43]. Currently in orthopedic surgeries, cephalosporins, aminoglycosides, quinolones and glycopeptide antibiotics have been widely used to prevent or treat infections [44]. The most of the antimicrobial drugs are active against these strains, but biofilm bacteria are extremely resistant to treatment with the conventional medication [2,32,45]. Overuse and misuse of these medications in medicine, food industry and agriculture have led to the appearance of multidrug-resistant, extensively drug-resistant and pan-drug-resistant strains [24,44,46,47,48,49].

Development of innovative biomaterials that focus on inhibition of both bacterial adhesion and biofilm formation is still a concern for many researchers [30,31]. In the opinion of several epidemiologists, antibiotic-releasing biomaterials may contribute to the selection and spreading of multidrug-resistant microorganisms [9]. Current strategies used in order to prevent the implant-associated infections, involve coating with antiseptics, antimicrobial polymers, metal ions or organic molecules [30,50,51,52,53]. The efficiency of coating depends on the clinical applications and device configuration [53]. Biomaterials with prolonged antibacterial activity were first proposed in the early 1950s in dentistry [54]. Afterwards, the interest to obtain carriers which could distribute active drugs directly at the site of infection was gradually extended to resorbable and even to soluble biomedical polymers [55].

A number of physical, chemical and biologic methods are practiced to achieve surface bioactivation, in order to reduce bacterial adhesion and improve biocompatibility [1,35].

## 3. Surface Functionalization with Antimicrobial Peptides

The emergence of antibiotic resistance affecting the human, animal and environment health is one of the world’s most urgent general public health problem [47,56,57,58,59,60]. Consequently, finding alternative therapies strategies is desirable to overcome and treat biofilm-based infections [38]. Therefore, natural antimicrobial peptides (AMPs) and their synthetic derivatives have acquired considerable attention as effective agents in various pathologies [61] through their broad spectrum of activity against bacterial (Gram-negative and Gram-positive bacteria, including drug resistant strains) and fungal microorganisms associated with low toxicity to mammalian cells, small molecular size and high stability [62,63,64].

Antimicrobial-peptide-based therapies are a substitute for antibiotic treatments, and offer several potential advantages [65]. Their antimicrobial mechanisms of AMPs are different from traditional antibiotics and have been linked to their structures [49,66]. It is generally accepted that the electrostatic interactions that occur between an AMP and the target cell’s membrane are the first step in their action and also present reduced bacterial resistance [67,68,69]. Based on their final effect on the target cells membranes, AMPs can be separated into two major classes: membrane disruptive AMPs and non-membrane disruptive AMPs [70]. However, resistance to AMPs may occur. Several mechanisms are recognized such as: cell envelope alteration, proteolytic degradation of the peptides, upregulation of efflux pumps and impedance by exopolymers and biofilm matrix molecules [24,71,72,73]. Due to externally applied AMPs resistance, cross-resistance to host AMPs or antimicrobial therapy may occur. The use of synthetic AMPs can prevent resistance to natural host defense peptides (HDPs) [48]. AMPs interrelate with certain specific constituents of the bacterial plasma membrane resulting in depolarization, destabilization, and/or disruption, leading to bacterial cell destruction [24]. AMPs are the host defense, naturally occurring peptides [33,49] and have been revealed to exhibit a broad spectrum of activities against Gram-positive and Gram-negative bacteria, fungi and viruses [61,74]. They have the capacity to neutralize virulence factors released by pathogens and also modulate the host immune response [75,76] and can also effectively attack bacteria within a biofilm (Ageitos et al. 2016).

More than 2700 different AMPs have been described to date [33,49]. Bacteriocins are bacterial AMPs, classified into class I bacteriocins known lantibiotics and class II bacteriocin/non-lantibiotics [77]. Lantibiotics contain lanthionine and 3-methyllanthionine and are produced by Gram-positive bacteria [78], while non-lantibiotics contain non-modified peptides or peptides with slight modifications [79]. Crucial conserved components of the innate immune system AMPs are the first-line defense against invading microorganisms [7,48,49,80]. AMPs, part of the innate immunity in a wide variety of organisms, with cationic and amphiphilic characteristics and well-defined hydrophobic and hydrophilic regions [7,48,49,80], are able to augment phagocytosis, stimulate prostaglandin release, neutralize the septic effects of lipopolysaccharides, promote angiogenesis and accumulation of various immune cells at inflammatory sites [81].

These peptides can be classified in two major antimicrobial types, based on amino acid composition structures and their biologic functions [74,80]. The collection of all known AMPs (more than 3000) can be found in the Antimicrobial Peptide Database (http://aps.unmc.edu/AP/main.php), but only seven small AMPs have been approved by the U.S. Food and Drug Administration (FDA) [82]. In the first subfamily there are included AMPs with linear molecules, α-helical structure and without cysteine, the second subfamily consists of cysteine-containing polypeptides [74]. Cathelicidins and defensins are two subfamilies derived from mammals. Cathelicidins are stored in the secretory granules of neutrophils and macrophages; their release is controlled by leukocyte activation [83]. Defensins are small cyclic peptides purified from granulocytes which are categorized into three subfamilies α-, β- and θ-defensins [84,85].

Generally, natural AMPs are not stable; for clinical applications it is essential to synthesize the long-acting peptide analogs [49]. The mechanism of action of synthetic AMPs involves inhibition of adherence of the bacteria to surfaces and/or reduction of expression of genes related to biofilm formation [86]. Cationic charge and peptidic nature of synthetic AMPs are a challenge for their biologic potential and antimicrobial efficacy [24].

The increase incidence of antibiotic resistance has stimulated the application of AMPs to medical devices [63]. Engineering biomaterial surfaces that include AMPs properties represent a hopeful approach to obviate implant infections [61]. Townsend and coworkers [63] reported the dual coating of the hydroxyapatite surface with AMPs using two different binding mechanisms. The covalently bonded peptide inhibits biofilm formation and the electrostatically released peptide inhibits bacterial growth [63]. AMPs are effective against a broad spectrum of microorganisms and also can work synergistically with classical antibiotics [61] in order to prevent the colonization of bacteria. Integration of AMPs into different type of carriers as a substitute of antimicrobials or in combination with antibiotics seems to be a hopeful approach for prevention or combating the bone infections [87]. Hydroxyapatite, chitosan, hyaluronic acid, polymethylmethacrylate in combination with various antibiotics has been extremely widely studied [36,88] for prevention of implant related infection. After implantation in organism there is a competition for implant surface (‘race to the surface’) between host cells involved in regeneration and pathogenic bacteria [36]. In order to prevent implant-associated infections several antimicrobial biomaterials with benefits and also with limitations have been developed such as antifouling surfaces, contact-killing surfaces and antimicrobial-releasing surfaces [24,89].

## 4. The Multiple Properties of Lactoferrin

**Figure 1:**
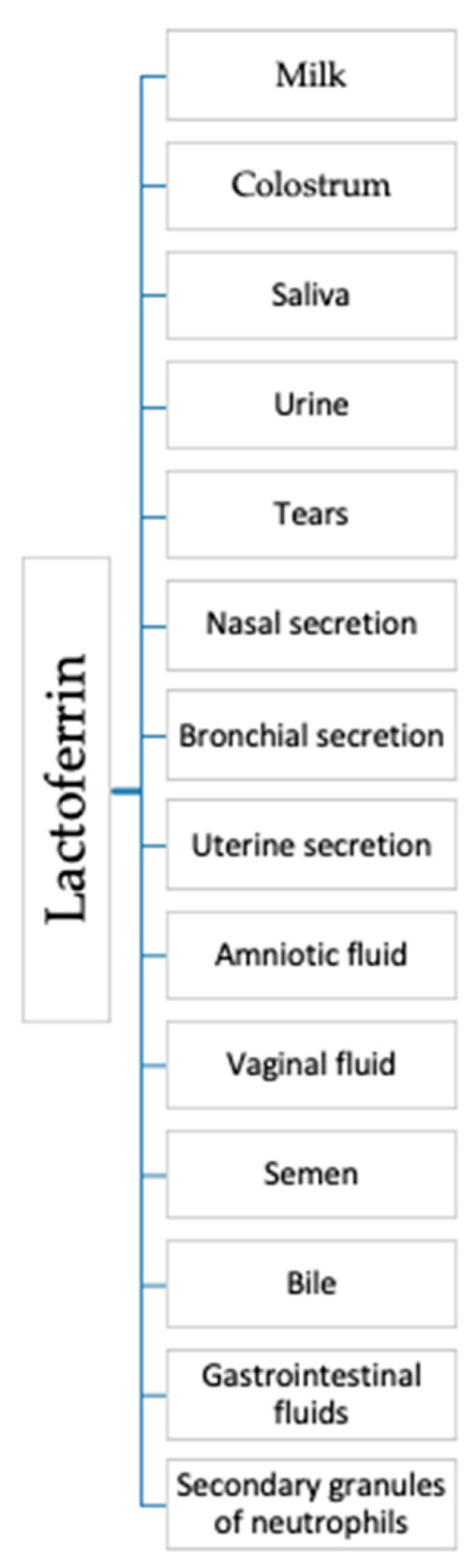
Sources of lactoferrin.

Lactoferrin (Lf) is a bioactive globular protein, belonging to the transferrin family, produced by epithelial cells and neutrophils in various mammalian species [90,91,92]. In healthy organisms lactoferrin is predominantly neutrophil-derived and is at a concentration of 2–7 × 10^−6^ g/mL [93]. Lf is an 80 kDa iron-binding multifunctional glycoprotein, source of cationic and hydrophobic antimicrobial peptides, found in most of the exocrine secretions such as milk, colostrum, saliva, urine, tears, nasal and bronchial secretions, uterine secretions, amniotic fluids, vaginal fluids, semen, bile and gastrointestinal fluids and also in secondary granules of neutrophils (Figure 1) [90,91,94].

**Figure 2:**
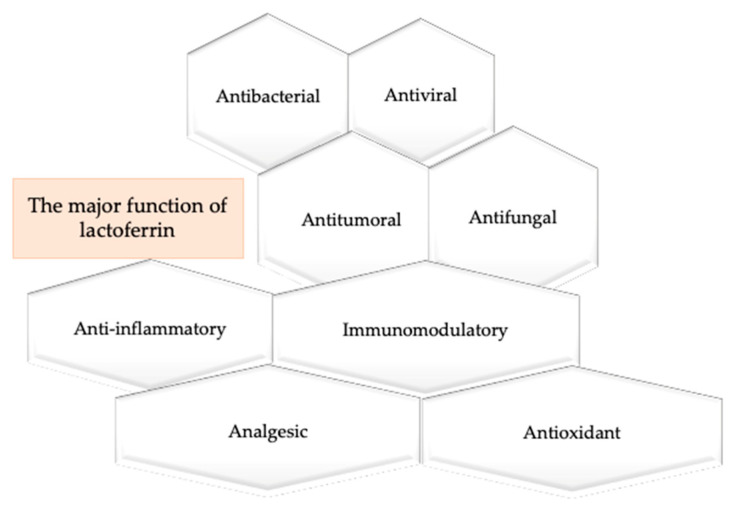
Major functions of lactoferrin.

Lf is a first-line defense protein [95] which possesses a range of biologic functions such as antibacterial, antiviral against a broad range of RNA and DNA viruses, antitumor, antifungal, anti-inflammatory, immunomodulatory, analgesic, antioxidant property (Figure 2).

Lf has a specific role in enhancement of lipid metabolism and can promote cytokine and chemokine production [91,95,96]. Biomimetic hydroxyapatite crystals, nanocrystals, biogenic silica surfaces functionalized with bioactive molecules like Lf, play an important role in various applications, including medicine, pharmacy, nanodevices, biosensors, bioengineering and regenerative therapy [57,97]. The antimicrobial properties of Lf are the most studied. Several mechanisms are involved in this activity such as iron chelation and thereby depriving microorganisms of this nutrient or direct interaction with bacterial surfaces components [90]. Bactericidal property of Lf it can also occurs through direct interaction with bacterial surfaces, with the change of membrane permeability, loss of cellular contents [98], followed by lysis, with the release of lipopolysaccharide [57,99,100,101] the outer membrane component of Gram-negative bacteria [91]. In Gram-positive bacteria, the mechanism of action of Lf is different; the bacterial membranes are disrupted by cationic residues and hydrophobic residues in the N-terminus [102]. Lf is a rich source of cationic and hydrophobic antimicrobial peptides and several studies show that Lf can neutralize the effect of lipopolysaccharide generated toxicity [91,103]. Lf interaction with lipopolysaccharide or with other bacterial membrane proteins increase the effect of natural bactericides such as lysozymes [101,104]. Lf can also modify and degrade virulence factors through proteolysis [102]. Yen et al. demonstrated that Lf is an effective bioactive protein in the prevention and treatment of infections with pathogens and multidrug-resistant bacterial strains [98,105]. In vitro studies confirmed that lactoferrin inhibits biofilm formation and disrupts existing biofilms [106].

The bioavailability of Lf in vivo is poor; contact with proteolytic enzymes [96,107] results in the production of antimicrobial peptides with superior potency than the native lactoferrin [91].

Stabilization can be achieved by incorporation of Lf into collagen-based biomaterials, hydrogels, liposomes, porous microspheres—or coating the surfaces of different types of implants [107]. Lf with anti-apoptotic effects can also modulate cell migration, adhesion, proliferation and osteogenic differentiation and potently inhibit osteoclastogenesis [108,109]. Lf also induces activation of p42/44 MAPK signaling in primary osteoblasts [93]. The work of Icriverzi and coworkers reported the osteoconductive and osteoinductive properties of Lf and hydroxyapatite loaded PEG–PCL biodegradable [110]. Collagen membranes treated with Lf also stimulate and promote osteogenic lineage differentiation [94,111]. In rat calvaria, defect gelatin hydrogel treated with Lf stimulates bone regeneration [112]. In human-adipose-tissue-derived stem cells Lf stimulate the synthesis of osteogenic differentiation-related marker genes [111]. Collagen–lactoferrin fibrillar coatings stimulate cells proliferation and differentiation and rapid bone healing [94]. In surgically created bone defects gelatin microspheres loaded with 3 mg of bovine lactoferrin in combination with anorganic bovine bone promotes bone regeneration [96]. The osteoblast differentiation potency of Lf was also demonstrated using a bone nodule formation assay. The results of study of Cornish et al. [112] demonstrated that a concentration of over 100 μg of Lf significantly stimulated the number of nodules and increased the mineralization. The study also investigated the feasibility of developing rhLF as a biomaterial for cell delivery [113].

Amini and Nair demonstrated anti-apoptotic effect of rhLF on MC3T3 pre-osteoblast cells, mechanism mediated by Wnt5a/PKA pathway. They also developed injectable matrix from rhLF which support cell viability, proliferation and differentiation [92].

Montesi et al. demonstrated that biomimetic hydroxyapatite nanocrystals have synergic behavior on bone homeostasis and also act as a potent anabolic factor for osteogenic differentiation and exhibit an inhibitor potential on osteoclast formation and activity [114]. Onasi et al. synthesized a chitosan–alginate–Ca microparticles in which they encapsulated Lf. These microcapsules have demonstrated better anti-inflammatory properties compared to free lactoferrin [115]. Kilic et al. first demonstrated the potential of layer-by-layer assembled multilayer microcapsules with bovine serum albumin, tannic acid and Lf [97]. Shi et al. developed two kinds of hydroxyapatites conjugated with lactoferrin. The maximum adsorption capacity of nano-hydroxyapatite is greater than that of micro- hydroxyapatites, due the larger surface area of nano-hydroxyapatite. They demonstrated that lactoferrin on hydroxyapatite surface could improve the biologic activity of hydroxyapatite [116]. Hydroxyapatite nanocrystals have been successfully used to fabricate bone scaffolds and implant coating materials and vehicles for drug targeting [117]. James et al. developed biodegradable hybrid polymeric nanofibrous scaffolds loaded with human recombinant lactoferrin [118] which demonstrated stimulating potential on MC3T3-E1 osteoblast-like cells adhesion and proliferation. The multifunctional character of Lf is assigned to a number of peptides derivatives such as lactoferrampin, lactoferricin with demonstrated effectiveness against *Candida albicans* and *Pseudomonas aeruginosa* [106]. Bolscher et al. studies indicated a mild antimicrobial property for lactoferrampin and enhanced antimicrobial efficacy for lactoferricin [119]. Singh et al. demonstrated that Lf prevent biofilm formation and disrupts existing biofilms, [120]. They used P. aeruginosa expressing green fluorescent protein (GFP) in continuous-culture-flow cells. In medium with Lf biofilm development was disrupted. The results of the study demonstrated the bactericidal and bacteriostatic actions of Lf against *P. aeruginosa*. Chen et al. [121] demonstrated that glass surfaces covalently bound with lactoferrin or lactoferricin can neutralize the microorganisms like *S. aureus* and *P. aeruginosa*. Fulgione et al. also demonstrated the therapeutic potential of lactoferrin delivered by biomimetic hydroxyapatite in bacterial infections [122]. Stoleru et al. successfully functionalized a poly(lactic acid) substrate with plasma or gamma irradiation and further anchoring with lactoferrin by covalent coupling using carbodiimide chemistry. This complex presented higher antioxidant, antimicrobial and cell-proliferation activity [123].

Jinkyu et al. developed a new electrospun nanofibers immobilized with lactoferrin by polydopamine chemistry with simultaneously anti-inflammatory and bone regeneration [124].

Godoy-Gallardo et al. demonstrated that the immobilization of hLf1-11 (GRRRRSVQWCA) peptide by silanization or through grafting of polymer brushes by surface-initiated polymerization significantly reduced bacterial adhesion and biofilm formation of S*treptococcus sanguinis* and *Lactobacillus salivarius* [125]. Costa et al. reported the covalent immobilization using specific orientation through its C-terminal cysteine of the hLF1–11 peptide onto chitosan ultrathin films. The functionalization of chitosan with hLF1–11 was able to attract and bind bacteria [126,127] (Table 1). Achievable applications for hLF (1–11) are to treat the surfaces of the medical devices as a nonaggressive bio disinfectant to inhibit adherence of bacteria and biofilm formation [127]. Nagano-Takebe et al. demonstrated that adsorbed human Lf on titanium-based biomaterial inhibited *Streptococcus gordonii* adhesion and also exhibited bactericidal activity [128]. Yoshinari et al. indicated that the modification of Ti surface with titanium binding peptides (minTBP-1) and lactoferricin leads to a reduction in the bioactivity of *Porphyromonas* gingivalis [129].

A major issue refers to the ability of biomaterials to release antimicrobials at the site of implantation. The ability of biomaterials to release antimicrobials at the site of implantation and or infection has been broadly studied [36]. Combinations of biomaterials with Lf or other type of AMPs could be a promising component of bone-implant materials solution to combat the problems and to eradicate multi drug resistant bacteria. A broad spectrum of compounds and technological approaches has been proposed, but for the expected effects it is important to establish their biocompatibility, antimicrobial efficiency and durability.

## 5. Conclusions

Several new studies have shown that prevention is the most favorable response to the problem of implants associated infections. The treatment of these infections is difficult, because the surface of the implant serves as a substrate for the formation of the biofilm and also for the selection of multidrug-resistant bacterial strains. The possibility to modulate the surfaces of the implants with different substances with antimicrobial effect has demonstrated efficacy and is considered a field with multiple potentials. Therefore, a promising new approach for prevention of implant-related infection involves the use of antimicrobial peptides with promising biologic effects in the treatment of various pathologies.

## Figures and Tables

**Table 1:** Biologic activity of Lf-coated biopolymers.

Material Description	Biologic Activity	Reference
Hydroxyapatite loaded PEG–PCL and lactoferrin	Osteoconductive and osteoinductive properties	[101]
Collagen membranes treated with Lf	Osteogenic lineage differentiation cells proliferation and differentiation	[94,91]
Gelatin hydrogel treated with Lf	Bone regeneration	[102]
AMSCs treatment with Lf	Osteogenic differentiation-related marker genes	[91]
Inorganic bovine bone and lactoferrin	Bone regeneration	[96]
rhLF on MC3T3 pre-osteoblast cells	Anti-apoptotic effect, support cell viability, proliferation and differentiation	[92]
Chitosan–alginate–Ca microparticles with Lf	Anti-inflammatory properties	[105,117]
Multilayer microcapsules with bovine serum albumin, tannic acid and Lf	Anti-inflammatory properties	[96]
Hydroxyapatites conjugated with lactoferrin	Antimicrobial property	[105]
Glass surfaces covalently bound with lactoferrin	Antimicrobial and antibiofilm property	[111]
Biopolymer loaded with hLf1-11	Antimicrobial and antibiofilm property	[112]
Chitosan ultrathin films with hLF1–11 peptide	Antimicrobial property	[113,114]
Human Lf on titanium-based biomaterial	Antimicrobial property	[115,116]
Biomimetic hydroxyapatite	Antimicrobial property	[122]
Functionalized the poly (lactic acid) substrate anchoring with lactoferrin	Antioxidant, antimicrobial and cell-proliferation activity	[123]
Electrospun nanofibers immobilized with lactoferrin	Anti-inflammatory and bone regeneration	[124]

PEG–PCL—poly(ethylene glycol)-poly(ε-caprolactone); Lf—lactoferrin; AMSCs—adipose-tissue-derived mesenchymal stem cells; rhLF—recombinant human lactoferrin; hLF1–11—human lactoferrin derived peptide.
